# Changes in Quality Characteristics, Microbial Communities, and Metabolomic Profiles of Mianning Ham During Different Aging Periods

**DOI:** 10.3390/foods15162831

**Published:** 2026-08-14

**Authors:** Yuejia Deng, Zhenghao Wang, Jiaxin Han, Lin Zhou, Xinhui Wang, Jing Zhang, Bingliang Liu, Weijun Chen

**Affiliations:** Meat Processing Key Laboratory of Sichuan Province, Food Security Publicity and Education Base of Sichuan Province, College of Food and Biological Engineering, Chengdu University, Chengdu 610106, China; dengyj20211001@126.com (Y.D.); w19113305889@163.com (Z.W.); 15877423632@163.com (J.H.); zhoulin94@cud.edu.cn (L.Z.); wangxinhui@cdu.edu.cn (X.W.); zhangjing2022@cdu.edu.cn (J.Z.)

**Keywords:** Mianning ham, aging period, microbial community, metabolomics

## Abstract

Mianning ham is a traditional dry-cured ham from southwestern China, but the coordinated relationships among quality changes, microbial succession, and nonvolatile metabolite remodeling during long-term ripening remain poorly understood. Therefore, samples of Mianning ham aged for one, two, three, and four years were comparatively analyzed. High-throughput sequencing was used to characterize microbial community composition, and untargeted metabolomics was applied to analyze differential metabolite changes and their associations with dominant microorganisms. The results showed that the physicochemical and sensory characteristics of Mianning ham exhibited stage-dependent changes during ripening, with moisture content and pH generally decreasing, while color, texture, and mature flavor characteristics gradually developed. Multivariate statistical analysis identified 99 differential metabolites, which were mainly involved in peptide accumulation, amino acid metabolism, and nitrogen-containing compound transformation. The bacterial communities were mainly composed of Bacillota and Pseudomonadota, while Ascomycota was the predominant fungal phylum. Distinct successional patterns of dominant microbial taxa were observed at different ripening stages. Furthermore, microorganism–metabolite correlation analysis revealed distinct association patterns between internal and surface samples of the ham. Internal microbial communities were mainly associated with nucleotide-related metabolism- and umami-related characteristics, whereas surface microbial communities were more closely associated with the transformation of protein-derived nitrogen-containing compounds and the accumulation of flavor precursors. This study provides insights into the quality formation patterns of Mianning ham during ripening and offers a theoretical basis for ripening-stage evaluation and quality regulation.

## 1. Introduction

Mianning ham, a traditional meat product from Liangshan Prefecture, Sichuan Province, is recognized as a National Geographical Indication agricultural product in China [1]. Its history can be traced back to the Ming Dynasty, indicating a production history of more than 500 years. Mianning ham is typically produced from the hind legs of Wujin pigs or crossbreeds of Wujin, Landrace, and Yorkshire pigs. During ripening, water loss, endogenous enzymatic activities, and microbial metabolism jointly participate in protein and lipid degradation processes and promote the formation of flavor-related metabolites. These changes gradually contribute to the development of the characteristic texture, taste, and aroma of Mianning ham.

The quality of dry-cured ham is influenced by a complex interplay among raw materials, processing practices, environmental conditions, and ripening time. Among these factors, ripening time is particularly important because physicochemical properties, microbial communities, and metabolite profiles continuously change during ripening.

Recent multiomics research across Jinhua, Xuanwei, and Panxian ham demonstrated that both production region and ripening time shape microbial, metabolomic, and volatilomic profiles [2]. In Nuodeng ham, metabolomics and gas chromatography revealed stage-dependent changes in small-molecule metabolites, free fatty acids, and characteristic volatiles over one to three years [3]. A complementary sequencing and GC-TOF-MS study further linked ripening-related microbial shifts with the accumulation of nonvolatile metabolites in the same product [4]. These findings indicate that ham quality emerges from coordinated biochemical and ecological changes rather than from any single quality indicator.

High-throughput sequencing and metabolomics have increasingly been combined to characterize the microbial and biochemical changes associated with dry-cured ham quality. In Xuanwei ham, integrated profiling identified significant associations among bacterial and fungal communities, nonvolatile metabolites, and volatile compounds [5]. Curing-agent studies in Nuodeng ham likewise showed that processing conditions can reshape bacterial communities and metabolite profiles [6]. However, previous studies of Mianning ham have mainly focused on microbial community characteristics and volatile flavor compounds [7,8]. The temporal relationships among bacterial and fungal succession, nonvolatile metabolite changes, and physicochemical and sensory properties during long-term ripening remain poorly understood. Moreover, few studies have investigated these quality-related characteristics simultaneously across multiple consecutive ripening years. This limitation hinders a comprehensive understanding of quality formation during Mianning ham ripening.

Therefore, the present study integrated quality measurements, high-throughput sequencing, and untargeted metabolomics to investigate Mianning ham samples after one, two, three, and four years of aging. The changes in physicochemical and sensory properties, bacterial and fungal community succession, and nonvolatile metabolite profiles during ripening were characterized, and potential associations between key microorganisms and differential metabolites were further explored using Spearman correlation analysis. This multi-dimensional and long-term study provides a systematic description of Mianning ham ripening and identifies microbial and metabolic features associated with quality development, providing scientific insights into quality formation and quality evaluation of Mianning ham.

## 2. Materials and Methods

### 2.1. Materials

The Mianning ham samples used in this study were obtained from Yuji Ham Food Co., Ltd. (Liangshan Yi Autonomous Prefecture, China). Methanol was supplied by Merck KGaA (Darmstadt, Germany), whereas chromatographic-grade acetonitrile and formic acid were sourced from Shanghai Xingke High-Purity Solvent Co., Ltd. (Shanghai, China). All chemicals were of analytical grade.

### 2.2. Sample Preparation

Ham processing began in October 2021, 2022, 2023, and 2024, respectively, and the main procedures included fresh leg trimming, salting, stacking and pressing, washing and shaping, hanging and air-drying, and aging. Different production batches were processed using the same procedures, and hind legs from pigs of similar age and body weight were selected as raw materials. The ripening process was managed according to the production hygiene requirements of Mianning ham. In October 2025, samples from different ripening stages were collected simultaneously. The production batches initiated in 2021, 2022, 2023, and 2024 corresponded to 4-, 3-, 2-, and 1-year ripening stages, respectively. Accordingly, HT1, HT2, HT3, and HT4 represented one-, two-, three-, and four-year-aged hams, respectively. Three independent hams were randomly selected from each ripening stage, resulting in a total of 12 ham samples.

Surface samples (HTS) and internal muscle samples (HTM) were collected for microbial community analysis. Surface samples were collected using sterile swabs. Three fixed sampling areas on the surface of each ham were uniformly swabbed, and the swabs were then transferred into sterile cryogenic tubes. Subsequently, the sampling areas and cutting instruments were disinfected with ethanol and sterilized by flame treatment. After removing the outer tissue using sterile knives, internal muscle tissues approximately 2 cm thick were aseptically excised from each ham, placed into sterile cryogenic tubes, and stored at −80 °C for subsequent microbial DNA extraction and community analysis. In addition, after removing subcutaneous fat and visible connective tissues, the biceps femoris muscle samples (HT) were collected for physicochemical analysis and metabolomics analysis.

### 2.3. Physicochemical Determinations

Moisture content was measured following the direct oven-drying procedure described by [9]. A 2 g portion of each sample was transferred to a drying oven (DGG-9070A; Shanghai Peiyin Experimental Instrument Co., Ltd., Shanghai, China) and dried at 105 °C to constant weight. pH measurements were conducted using a pH meter (testo 206; Testo SE & Co. KGaA, Titisee-Neustadt, Germany). Thiobarbituric acid reactive substances (TBARS) values were measured following the method described previously and expressed as mg MDA/kg [9]. Total volatile basic nitrogen (TVB-N) content was analyzed using a slightly modified semi-micro nitrogen assay procedure based on a previously reported method, and the values were reported as mg/100 g [10].

#### 2.3.1. Determination of Color

Color measurements were performed on the lean portion of the ham using a colorimeter (SC-10; Shenzhen 3nh Technology Co., Ltd., Shenzhen, China). Before analysis, the instrument was calibrated with a standard white plate. Three sites were randomly selected from each sample to determine lightness (L*), redness (a*), and yellowness (b*) values, and each site was measured in triplicate [10].

#### 2.3.2. Texture Profile Analysis (TPA)

TPA was performed using a texture analyzer (TA.TOUCH; BosinTech, Shanghai, China). Meat samples were excised as 10 × 10 × 10 mm cubes, with the cutting direction aligned along the orientation of the muscle fibers. Texture measurements were performed using a TA/36 probe, following the instrumental parameters described previously, with slight modifications: trigger type, Auto (Force); trigger force, 5.0 g; pre-test speed, 2.00 mm/s; test speed, 1.00 mm/s; post-test speed, 3.00 mm/s; strain, 40.0%; return height, 10 mm; and interval between two compressions, 5 s [11]. Triplicate measurements were conducted for each sample, and the values of hardness, springiness, cohesiveness, chewiness, and resilience were recorded.

### 2.4. Sensory Analysis

The sensory assessment was carried out using a modified procedure based on the method described previously [12]. Ten trained panelists from the College of Food and Biological Engineering, Chengdu University, participated in the evaluation, including five males and five females aged 20–30 years. Before the formal evaluation, all panelists received standardized training to familiarize themselves with the sensory attributes and evaluation criteria of ham samples. The training included sensory attribute definition, use of the scoring scale, identification of reference samples, and consistency training.

Ham samples were sliced into approximately 1 mm thick pieces, placed on white plastic trays, and randomly assigned three-digit codes before being presented to the panelists. The sensory evaluation was conducted under controlled environmental conditions to minimize the influence of external odors and other environmental factors on sensory perception. Samples were presented at room temperature in a randomized order. The panelists independently evaluated the samples for appearance (redness and color uniformity), aroma (fatty aroma and aged-meat aroma), taste (umami and saltiness), and texture (hardness and oiliness). A nonlinear structured scale from 0 to 7 was used, where 0 indicated that the attribute was not perceptible and 7 indicated its highest intensity. Drinking water and unsalted bread were provided during the evaluation for palate cleansing and taste recovery between samples.

### 2.5. Microbial Diversity Analysis

Total DNA was extracted from each sample using the Magnetic Soil and Stool DNA Kit (DP712; Tiangen Biotech Co., Ltd., Beijing, China). For bacterial profiling, 16S rRNA amplicons were generated using primers 341F (5′-CCTACGGGNGGCWGCAG-3′) and 805R (5′-GACTACHVGGGTATCTAATCC-3′), whereas fungal ITS1 amplicons were obtained using primers fITS7F (GTGARTCATCGAATCTTTG) and ITS4R (TCCTCCGCTTATTGATATGC). Qualified amplicon libraries were subsequently prepared and subjected to paired-end sequencing (2 × 250 bp) on the DNBSEQ-G99 platform by LC-Bio Technology Co., Ltd. (Hangzhou, China). QIIME 2 (version 2025.7) was used to perform read quality control, assemble paired-end sequences, and filter out chimeric reads. An amplicon sequence variant (ASV) table was then generated using DADA2, and taxonomic annotation was assigned based on the SILVA database (release 138.2).

### 2.6. Small-Molecule Metabolite Analysis

Ham samples were thawed on ice and pulverized under liquid nitrogen. For metabolite extraction, 20 mg of each sample was homogenized in 400 μL of methanol/water solution containing internal standards (methanol:water, 7:3, *v*/*v*), followed by shaking for 5 min and incubation on ice for 15 min. After centrifugation at 12,000 rpm for 10 min at 4 °C, the resulting supernatant was collected for liquid chromatography–mass spectrometry (LC–MS) analysis. Quality control (QC) samples were prepared by mixing equal volumes of extracts from all samples and were analyzed together with experimental samples to evaluate the stability and reproducibility of the LC–MS analysis. Mass spectra were collected in both positive and negative electrospray ionization modes during gradient elution. Chromatographic separation was performed using a Waters ACQUITY Premier HSS T3 column (1.8 μm, 2.1 mm × 100 mm), with 0.1% formic acid in water as mobile phase A and 0.1% formic acid in acetonitrile as mobile phase B. The column temperature was maintained at 40 °C, with a flow rate of 0.4 mL/min and an injection volume of 3 μL. Full-scan and data-dependent MS/MS acquisition were performed in both ionization modes across an m/z range of 70–1000. LC–MS raw data were processed using a standard untargeted metabolomics workflow, including peak detection, alignment, and normalization. Metabolite annotation was performed based on accurate mass matching and MS/MS fragmentation information.

### 2.7. Statistical Analysis

Data from the experiments were compiled in Microsoft Excel 2016 (Microsoft Corporation, Redmond, WA, USA) and analyzed by one-way analysis of variance (ANOVA) using IBM SPSS Statistics 27.0 (IBM SPSS Inc., Chicago, IL, USA). Post hoc comparisons were performed using Duncan’s test. Results are presented as mean ± standard error, with significance set at *p* < 0.05. Data visualization was performed using Origin 2024 (OriginLab Corporation, Northampton, MA, USA). Spearman correlation analysis was performed to evaluate the relationships between microorganisms and differential metabolites, with |r| ≥ 0.6 and *p* < 0.05 considered significant.

## 3. Results and Discussion

### 3.1. Analysis of Physicochemical Properties

**Figure 1:**
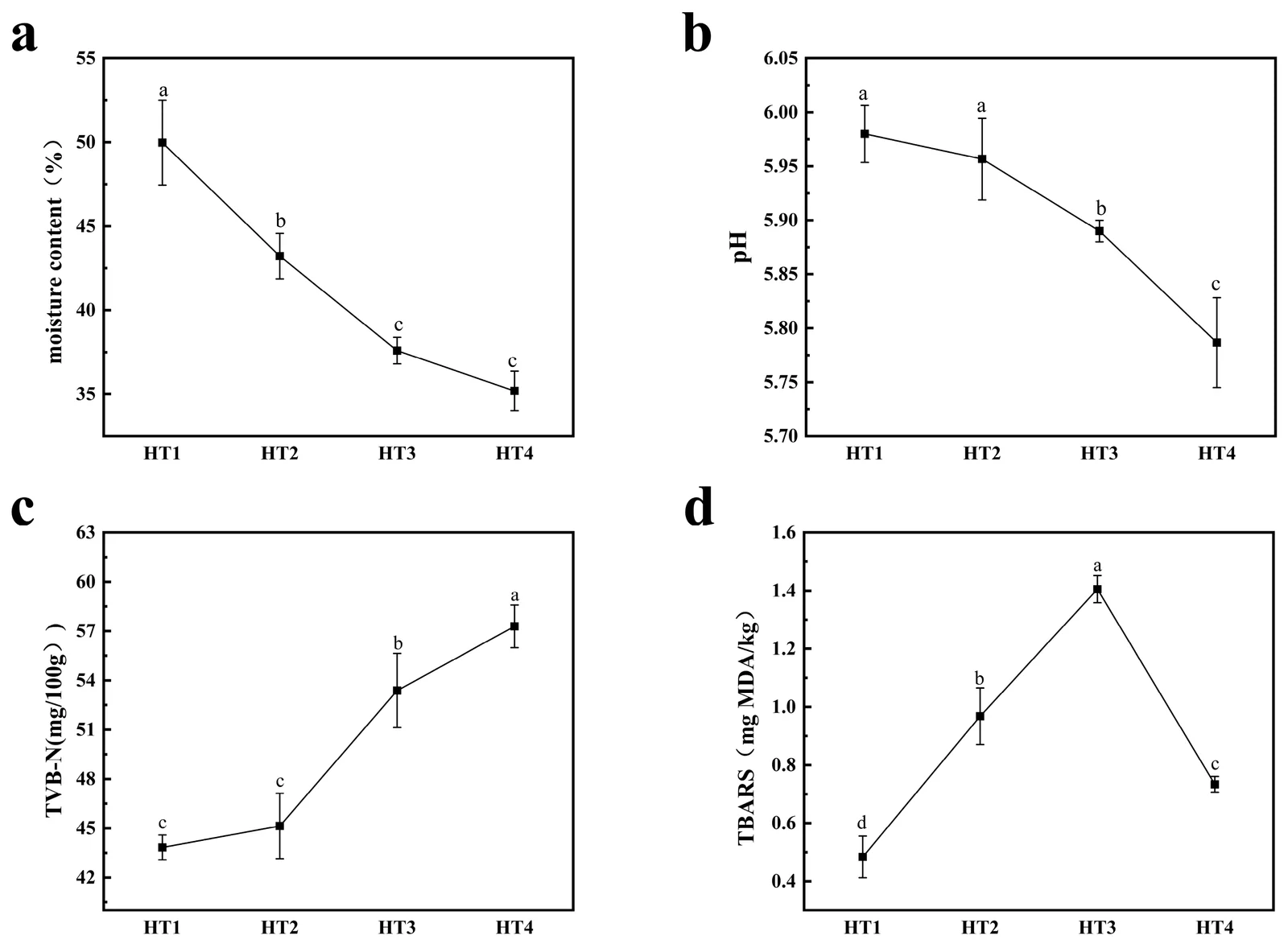
Physicochemical parameters of Mianning ham at different ripening years. HT1, HT2, HT3, and HT4 represent one-, two-, three-, and four-year-aged hams, respectively. Notes: (**a**) moisture content; (**b**) pH; (**c**) TVB-N; (**d**) TBARS. Different letters indicate statistically significant differences (*p* < 0.05).

Moisture is an important component of ham, and its content directly affects the taste and texture of the product [13]. Figure 1a shows that the moisture content of hams with different ripening periods decreased continuously with prolonged maturation. The one-year-aged ham exhibited the highest moisture content, at 49.97 g/100 g. Compared with the one-year-aged ham, the moisture contents of the two- and three-year-aged hams showed significant decreases of 6.75% and 12.38%, respectively (*p* < 0.05). The moisture content of the four-year-aged ham was also significantly lower than that of the one-year-aged ham (*p* < 0.05), a trend that has also been reported in related studies [5]. The decrease in moisture content during ripening was mainly attributed to continuous water migration and evaporation under long-term ripening conditions. With prolonged ripening, salt diffusion and changes in muscle tissue structure promoted gradual water loss, resulting in decreased moisture content of ham.

The pH value of dry-cured ham is a key factor determining texture, microbial safety, and biochemical stability [14]. Among Mianning hams aged for different durations, pH showed a decreasing trend, which may be associated with the accumulation of acidic substances caused by microbial metabolic activities during ripening. No significant variation in pH was found between the one- and two-year-aged hams, with values remaining stable at approximately 6.00. However, the pH values of the three- and four-year-aged hams decreased significantly (*p* < 0.05), reaching 5.89 and 5.78, respectively. These values were slightly higher than those reported in other studies [7]. The increased relative abundance of *Latilactobacillus* in three-year-aged ham may further contribute to the accumulation of acidic metabolites such as organic acids, which was consistent with the further decrease in pH value.

TVB-N is commonly used to reflect protein degradation and freshness variation in meat products. During prolonged ripening, enhanced protein hydrolysis, amino acid deamination, and microbial metabolic activities may promote the formation and accumulation of volatile basic nitrogen compounds [15]. Figure 1c shows that the TVB-N value gradually increased with prolonged ripening. The TVB-N values of the one- and two-year-aged hams were relatively low, approximately 44 mg/100 g, whereas those of the three- and four-year-aged hams increased significantly (*p* < 0.05). Nevertheless, the TVB-N content of the four-year-aged ham remained within the normal concentration range for ham products [16]. Meanwhile, changes in amino acid- and small peptide-related differential metabolites further supported the continuous degradation of proteins and transformation of nitrogen-containing compounds during ripening, which may contribute to the formation of volatile basic nitrogen compounds.

Lipids in dry-cured ham mainly include triacylglycerols and membrane lipids. During ripening, free fatty acids generated from lipid hydrolysis are susceptible to oxidation, producing oxidation products such as malondialdehyde, thereby contributing to increased TBARS values [17]. TBARS values are commonly used to indicate lipid oxidation and are widely applied to evaluate lipid oxidation levels in meat products. According to Figure 1d, the TBARS value showed an initial increase followed by a decline. The TBARS values increased annually from the one-year-aged- to the three-year-aged hams, reaching the highest value of 1.41 mg/kg in the three-year-aged ham. In contrast, the TBARS value of the four-year-aged ham was significantly reduced (*p* < 0.05), yet its level remained above that observed in the one-year-aged ham. This change may be associated with microbial community succession and related metabolic alterations during the late stage of ripening. Previous studies have shown that lactic acid bacteria fermentation of protein substrates can generate protein hydrolysates and small peptides with potential antioxidant activity [18]. Meanwhile, protein degradation and related metabolic processes continue during ham ripening, and the resulting small peptide degradation products may be associated with changes in lipid oxidation levels.

### 3.2. Sensory Analysis

Color, especially redness, is a key quality attribute of dry-cured ham and directly affects consumers’ purchase intention and sensory perception [17]. According to Table 1, the L* value of Mianning ham showed a significant decline during prolonged maturation (*p* < 0.05). The one-year-aged ham showed the highest L* value, at 29.67, which is consistent with the results reported in similar studies [19]. In contrast, the a* and b* values of Mianning ham gradually increased with prolonged aging. The four-year-aged ham had the highest a* and b* values, at 14.91 and 15.98, respectively, indicating that the ham became darker and showed more pronounced redness and yellowness as maturation progressed. The elevated a* value may result from the accumulation of stable red pigments, such as zinc protoporphyrin, which is closely related to the degradation of myoglobin and other heme proteins [20].

Texture is an important characteristic used to evaluate ham quality [12]. As shown in Table 1, the hardness of Mianning ham increased significantly with prolonged maturation. The hardness of the four-year-aged ham reached 10,818.68 gf, which was significantly higher than that of hams from the other ripening periods (*p* < 0.05). The springiness of the ham initially increased and subsequently decreased. The three-year-aged ham exhibited the highest springiness, at 0.67, which was 67.50% and 34.00% higher than that of the one- and two-year-aged hams, respectively (*p* < 0.05). The springiness of the four-year-aged ham decreased significantly (*p* < 0.05) but remained higher than that of the two-year-aged ham. Chewiness and resilience showed similar trends, both reaching their maximum values in the three-year-aged ham, at 3314.84 and 0.11, respectively. In contrast, the resilience of the four-year-aged ham was significantly lower than that of hams from the other ripening periods (*p* < 0.05). The trend in cohesiveness differed from those observed for the other texture parameters. The three-year-aged ham showed the lowest cohesiveness, at 0.38 (*p* < 0.05), whereas the two-year-aged ham had the highest cohesiveness, followed by the four-year-aged ham. Overall, the hardness of Mianning ham increased gradually with prolonged maturation, which was mainly related to salt accumulation and moisture loss during processing. These changes promote increased hardness and chewiness in dry-cured ham [21]. Springiness, chewiness, and resilience showed optimal values at the three-year-aged maturation stage, whereas changes in springiness and resilience may be associated with moisture loss and the disruption of myofibrillar structure [22].

**Figure 2:**
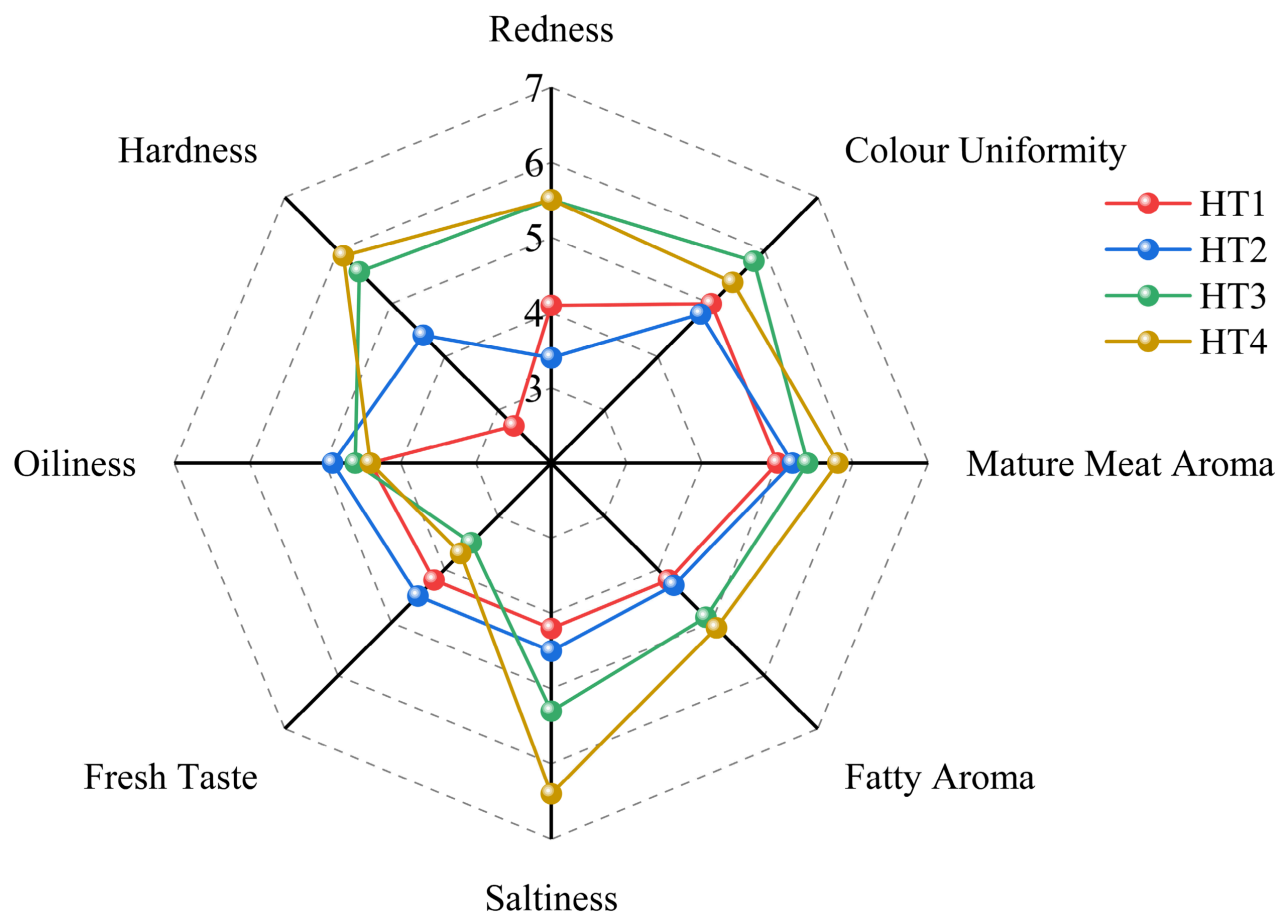
Radar chart of sensory evaluation of Mianning ham at different ripening years. Notes: The sensory attributes were evaluated on a 0–7 scale, where 0 indicates not perceptible and 7 indicates the highest intensity.

Figure 2 shows the radar chart of sensory evaluation for Mianning hams aged for different periods. In terms of appearance, the redness and color uniformity scores of the three- and four-year-aged hams were higher than those of the one- and two-year-aged hams, which is consistent with the instrumental color measurements. In terms of aroma, the four-year-aged ham had the highest scores for aged-meat aroma and fatty aroma, followed by the three-, two-, and one-year-aged hams. Higher salt content affects the formation of aldehydes, which are considered important aroma compounds in dry-cured ham [23]. Regarding mouthfeel, the four-year-aged ham had the highest hardness score, whereas the one-year-aged ham had the lowest. This may be attributed to increased salt content and moisture loss, which led to enhanced hardness and chewiness in dry-cured ham, consistent with the hardness results obtained from texture profile analysis [24].

### 3.3. Analysis of Small-Molecule Metabolites

#### 3.3.1. Multivariate Statistical Analysis of Small-Molecule Metabolites

**Figure 3:**
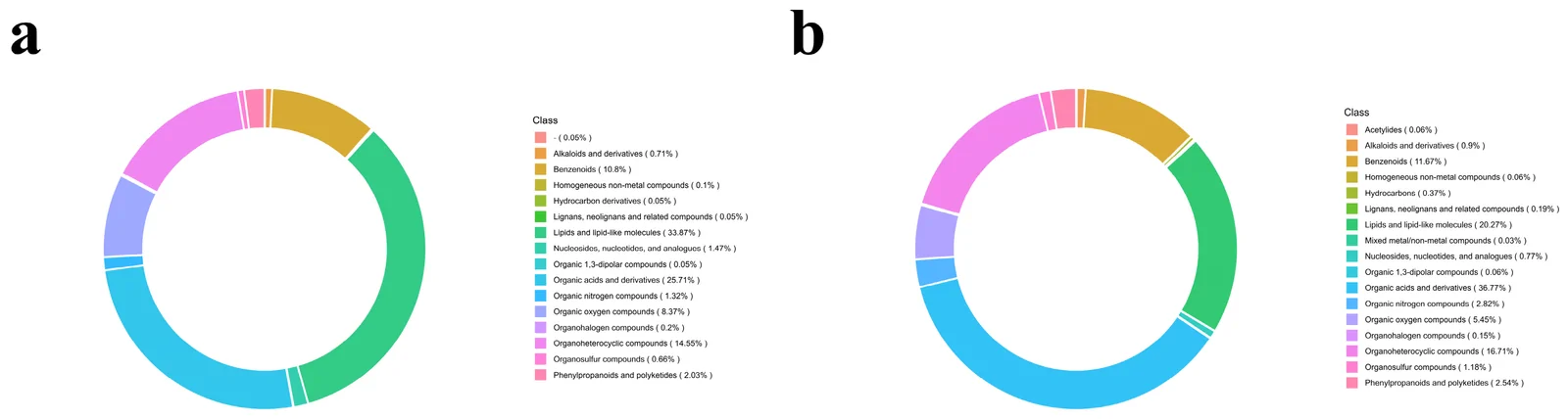
Metabolite class composition of Mianning ham. Notes: (**a**) positive ion mode; (**b**) negative ion mode. The “-” mark in figure (**a**) indicates unclassified compounds.

LC–MS analysis detected 22,887 and 1698 small-molecule metabolites in Mianning ham samples aged for different periods in positive and negative ion modes, respectively. Based on the HMDB database, these metabolites were classified into 18 categories. Figure 3a,b show the metabolite class compositions of Mianning ham samples in positive and negative ion modes, respectively. Lipids and lipid-like molecules, organic acids and derivatives, and organoheterocyclic compounds were the three most abundant metabolite categories.

**Figure 4:**
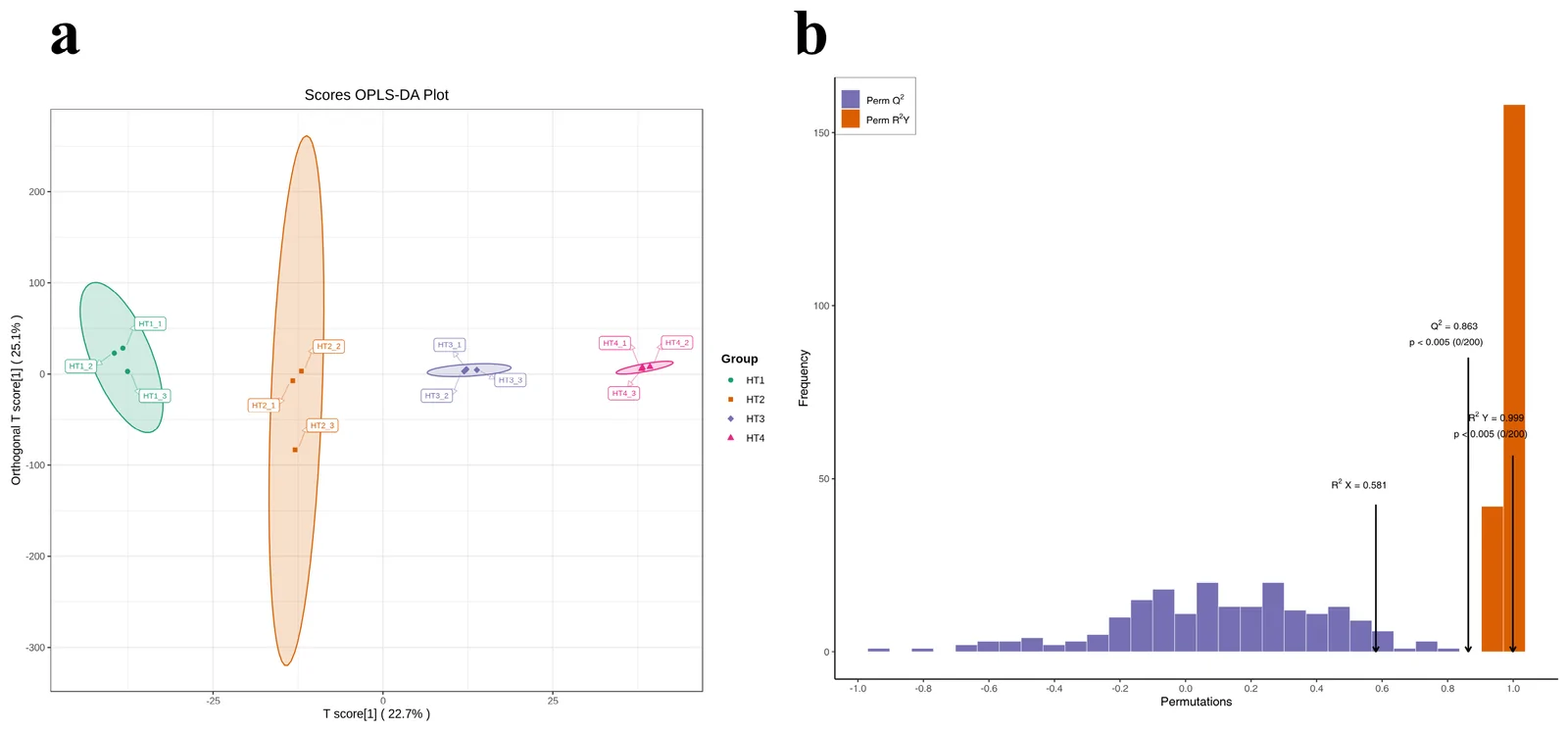
OPLS-DA. Notes: (**a**) OPLS-DA score plot; (**b**) permutation test plot of the OPLS-DA model.

Orthogonal partial least squares discriminant analysis (OPLS-DA) is a supervised multivariate statistical method that can effectively eliminate variations unrelated to the research objective, thereby facilitating the screening of differential metabolites. As shown in Figure 4a, clear separation was observed among the different comparison groups, indicating that the metabolic profiles of ham samples changed with ripening duration. In addition, the R^2^Y (0.999) and Q^2^ (0.863) values of the model were both greater than 0.5, suggesting that the model had good explanatory and predictive abilities. Therefore, the constructed model was considered reliable.

#### 3.3.2. Identification of Characteristic Metabolites

**Figure 5:**
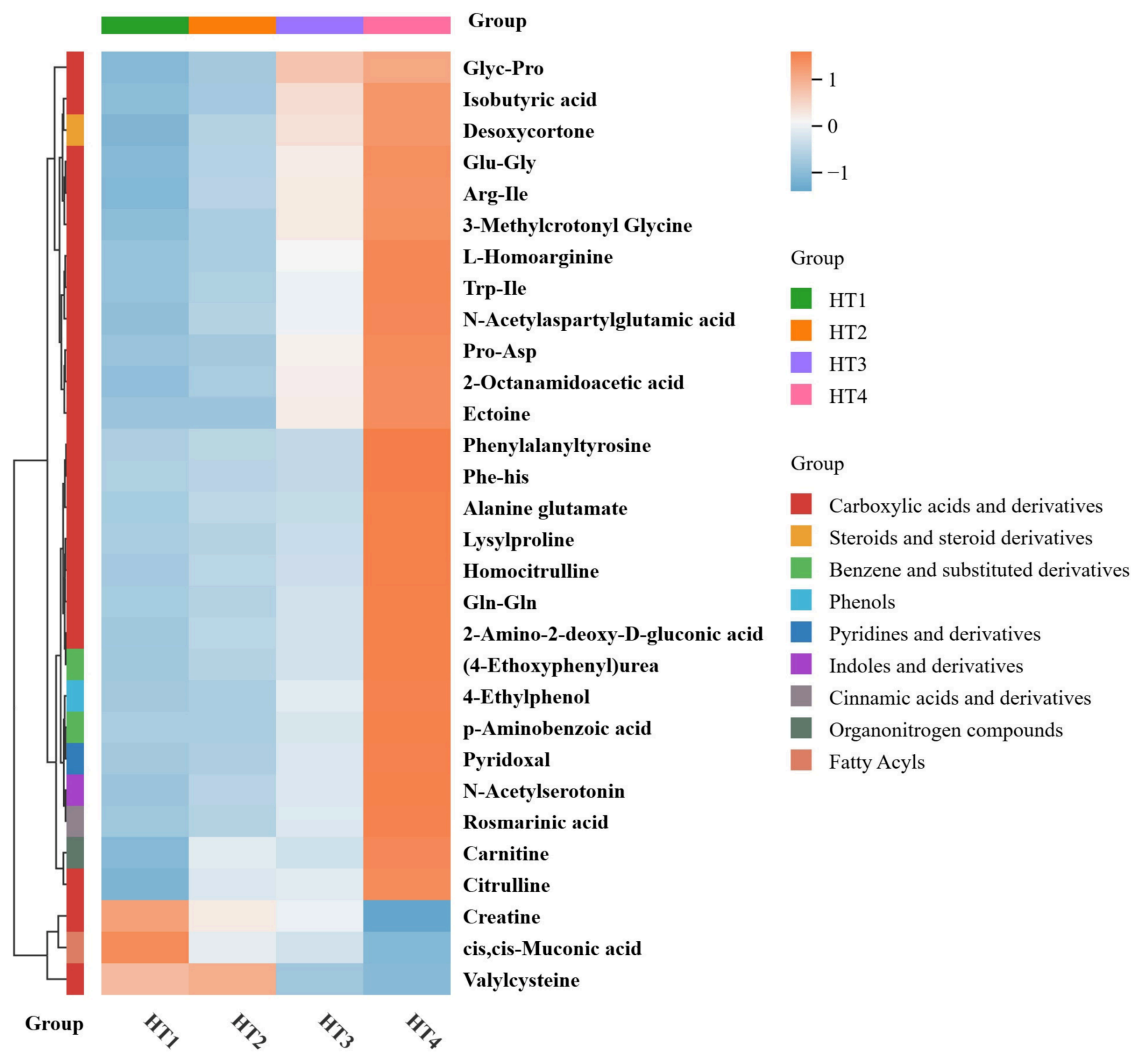
Clustered heat map of differential metabolites in Mianning ham at different ripening stages.

To elucidate metabolite changes in Mianning ham, differential metabolites were screened using VIP values > 1.5 from the OPLS-DA model and *p*-values < 0.05 as the criteria. A total of 99 differential metabolites were ultimately identified, among which carboxylic acids and their derivatives accounted for a relatively high proportion. Based on the ranking of VIP values, the top 30 differential metabolites were selected to construct a clustered heat map. As illustrated in Figure 5, metabolite relative abundance profiles in Mianning ham samples with different ripening periods exhibited distinct stage-specific distribution patterns. Overall, most characteristic differential metabolites showed relatively low abundance in the one- and two-year-aged samples, began to increase in the three-year-aged samples, and reached higher levels in the four-year-aged samples, indicating that some key metabolites gradually accumulated with prolonged ripening duration.

The differential metabolites could be broadly classified into two categories. The first category comprised continuously accumulated metabolites, which were characterized by gradual accumulation as ripening progressed and included Glyc-Pro, Glu-Gly, Arg-Ile, L-homoarginine, Trp-Ile, Pro-Asp, lysylproline, homocitrulline, Gln-Gln, carnitine, and citrulline. The second category comprised early-stage enriched metabolites, which showed relatively high abundance in the one-year-aged samples and then gradually decreased with prolonged ripening duration, including creatine, cis,cis-muconic acid, and valylcysteine. Creatine has been identified as a major differential bitter compound in Jinhua ham [16]. It is generally believed that, during the ripening of dry-cured ham, creatine can be gradually converted into creatinine. However, previous studies have pointed out that the creatinine/creatine ratio cannot be regarded as a biomarker of ripening duration [25]. In addition, some aromatic and heterocyclic metabolites, such as rosmarinic acid, *N*-acetylserotonin, 4-ethylphenol, and p-aminobenzoic acid, exhibited higher relative abundance in the four-year-aged samples, suggesting that, in the later stages of ripening, the accumulation of small peptides, amino acids, and their derivatives may be accompanied by the enrichment of aromatic metabolism-related products and potential flavor-active small molecules. In contrast, metabolites such as creatine and valylcysteine showed relatively high abundance in early-stage samples but decreased in later-stage samples, indicating that these compounds may be further degraded or participate in other metabolic pathways during ripening.

#### 3.3.3. KEGG Enrichment Analysis of Differential Metabolites

To characterize the metabolic routes associated with differential metabolites in Mianning ham across different ripening periods, pathway enrichment analysis was performed using the MetaboAnalyst 5.0 platform. A total of 19 metabolic pathways were annotated. Considering both the statistical significance of pathway enrichment and the pathway impact values obtained from topology analysis, pathways with an impact value greater than 0.01 were further selected, resulting in 14 potential key metabolic pathways.

**Figure 6:**
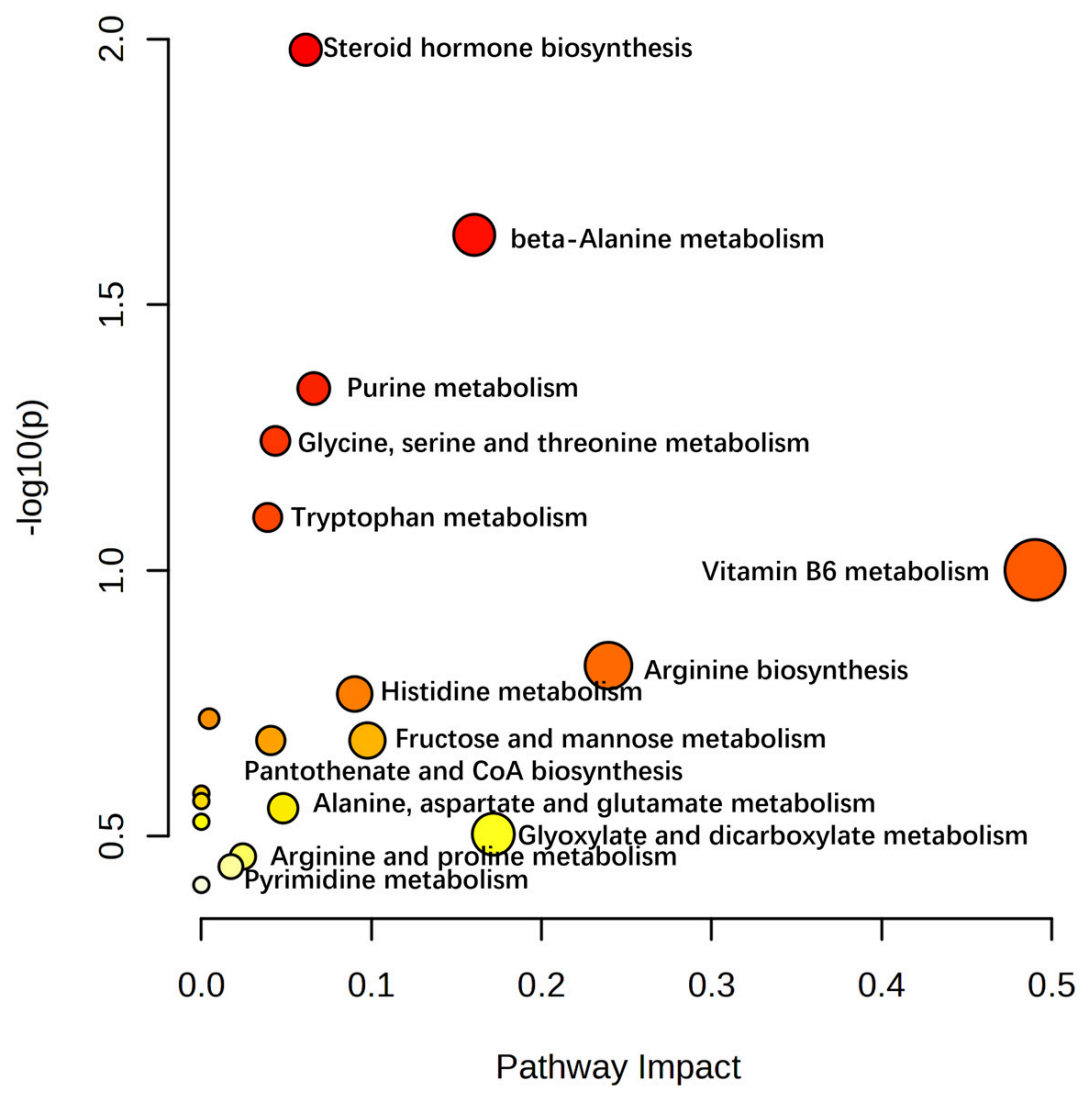
Bubble plot of metabolic pathway analysis of differential metabolites. Notes: Bubble color denotes statistical significance (red: highly significant; white: low significance), while bubble size reflects the pathway impact value.

According to the KEGG enrichment results shown in Figure 6, the differential metabolites were predominantly involved in glycine, serine and threonine metabolism, β-alanine metabolism, arginine biosynthesis, and histidine metabolism. Most of these pathways are directly involved in amino acid metabolism or are closely associated with amino acid conversion, suggesting that amino acid metabolism represents a major direction of metabolite remodeling during the ripening of Mianning ham. Specifically, glycine has a sweet taste, and its accumulation during ripening may help improve the mellowness and balance of ham flavor while also modulating undesirable tastes such as sourness and astringency [26]. Previous work has suggested that glycine may help control biogenic amine accumulation by inhibiting amine-producing bacteria and decreasing the activity of amino acid decarboxylases [27]. Serine and threonine are generally classified as sweet amino acids. Serine is also an important precursor in one-carbon metabolism and multiple biosynthetic reactions, linking metabolic processes such as glycolysis, purine synthesis, the one-carbon cycle, and glutathione synthesis and serving as an important hub in cellular carbon and nitrogen metabolism [28]. Arginine and histidine are generally associated with bitter taste, and histidine can be metabolically converted into histamine. The increased relative abundance of various small peptides, amino acids, and amino acid derivatives in samples ripened for longer periods indicates that protein degradation became more extensive as ripening progressed, accompanied by sustained enhancement of amino acid metabolism. These changes may further contribute to the balance among umami, sweet, and bitter taste attributes, while also promoting the accumulation of precursors related to mature flavor formation. Thus, amino acid metabolism is not only a major feature of differential metabolite variation, but also an important metabolic link connecting ripening duration, metabolite remodeling, and quality development in Mianning ham.

### 3.4. Analysis of Microbial Community Structure

#### 3.4.1. Analysis of Bacterial Diversity

The bacterial community structure of Mianning ham samples from different ripening years was characterized using high-throughput sequencing. As shown in Table 2, a total of 633,283 raw sequences were obtained by bacterial 16S rRNA gene sequencing. After paired-end assembly, removal of low-quality sequences, and chimera filtering, 564,674 high-quality sequences were retained. The numbers of raw and high-quality sequences in each sample were generally balanced, indicating that sequencing depth was largely consistent among samples and that the data quality was relatively high. Therefore, the sequencing data were suitable for subsequent analyses of community diversity and composition.

Alpha diversity refers to the diversity within a specific habitat and can be evaluated using indices such as ACE, Chao1, Shannon, and Simpson, which reflect community richness and diversity. According to Table S1, all Mianning ham samples showed coverage values above 99.00%, indicating that the sequencing depth adequately covered the dominant microbial taxa in the samples and was sufficient for subsequent diversity analysis.

Analysis of bacterial α-diversity in the internal samples of Mianning ham showed that the ACE and Chao1 indices of the one-year-aged ham were significantly higher than those of samples with other ripening years (*p* < 0.05). The Shannon and Simpson indices were also relatively high, indicating that the internal bacterial community of one-year-aged ham had higher richness and diversity. In contrast, four-year-aged ham had the lowest ACE and Chao1 indices, and its Shannon and Simpson indices were also significantly lower than those of hams from other years (*p* < 0.05), suggesting that both the richness and diversity of the internal bacterial community in four-year-aged ham were lower than those in hams from other ripening years. For the bacterial communities in surface samples, the ACE and Chao1 indices of four-year-aged ham were the highest (*p* < 0.05), indicating that the surface bacterial community of four-year-aged ham had the greatest richness. Meanwhile, the Shannon index of three-year-aged ham was significantly higher than those of one-, two-, and four-year-aged hams (*p* < 0.05), and its Simpson index was also the highest, suggesting that the surface bacterial community diversity of three-year-aged ham was the highest.

As shown in Figure S1, for internal samples of Mianning ham, the first two axes of principal coordinate analysis (PCoA), PCoA1 and PCoA2, explained 21% and 14% of the variation in bacterial community structure, respectively, with a total explained variance of 35%. Permutational multivariate analysis of variance (PERMANOVA) results (R^2^ = 0.3314, *p* = 0.052) indicated that differences in bacterial community structure among internal samples from different ripening years did not reach statistical significance. However, the *p*-value was close to 0.05, suggesting that ripening time may have some influence on the internal bacterial community structure of Mianning ham. For surface samples, PCoA1 and PCoA2 explained 41% and 29% of the variation in bacterial community structure, respectively, with a total explained variance of 70%. The PERMANOVA results (R^2^ = 0.4399, *p* = 0.023) indicated that ripening time had a significant effect on the bacterial community structure on the surface of Mianning ham.

#### 3.4.2. Bacterial Community Composition

**Figure 7:**
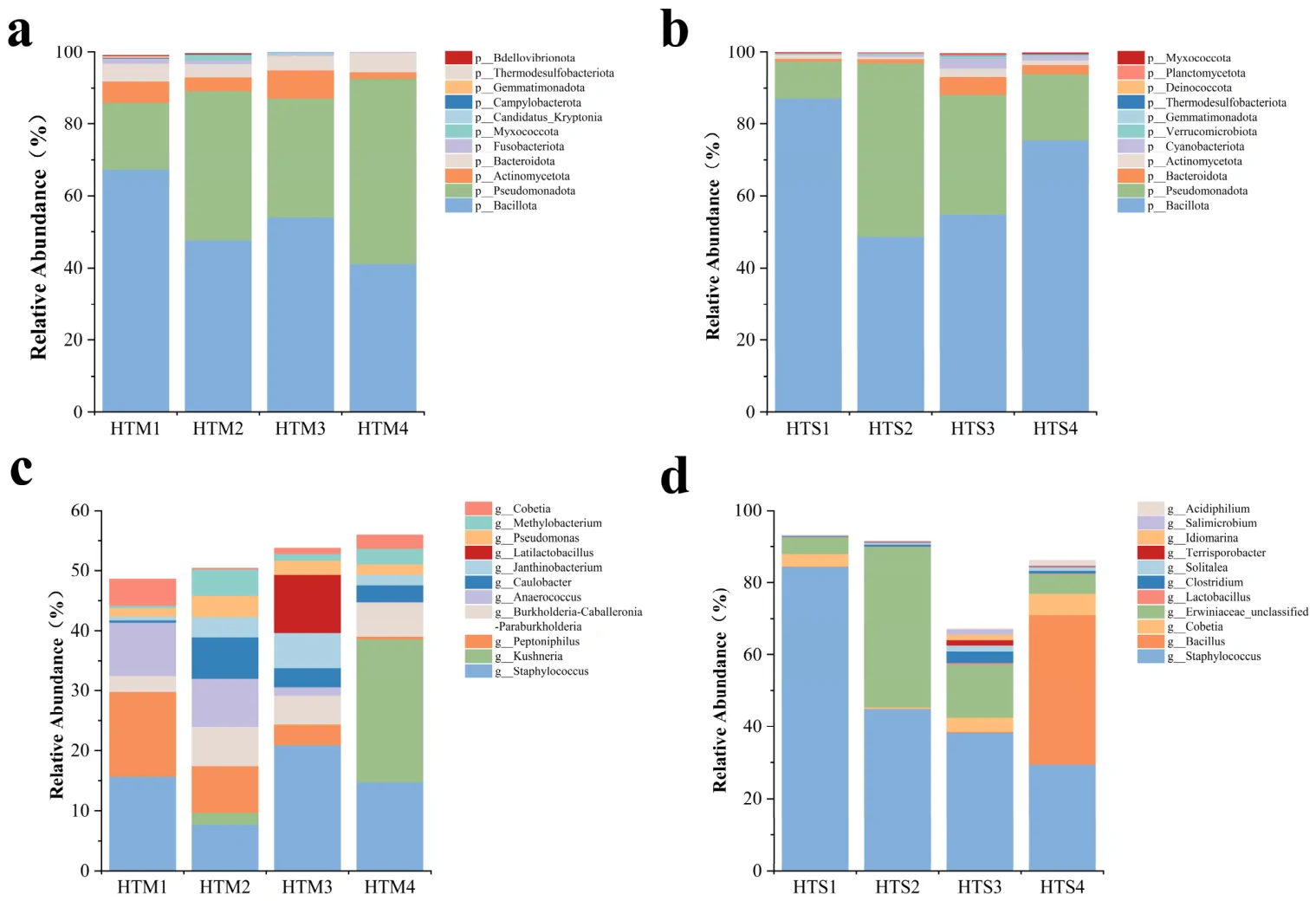
Stacked bar plots of the relative abundance of bacterial communities at the phylum and genus levels. Notes: (**a**) internal bacterial community at the phylum level; (**b**) surface bacterial community at the phylum level; (**c**) internal bacterial community at the genus level; (**d**) surface bacterial community at the genus level.

At the phylum level, Bacillota, Pseudomonadota, Actinomycetota, and Bacteroidota were the predominant bacterial groups detected in the internal Mianning ham samples (Figure 7), which is consistent with previous findings on dry-cured meat products [7]. Among these phyla, Bacillota was dominant, with the highest relative abundance observed in internal samples of one-year-aged ham, at 67.30%. With prolonged ripening, its relative abundance generally decreased, accounting for 47.85%, 54.08%, and 41.15% in internal samples of two-, three-, and four-year-aged hams, respectively. In contrast, the relative abundance of Pseudomonadota gradually increased in internal samples from two- to four-year-aged hams and became dominant in four-year-aged ham, reaching 51.47%. Previous studies have shown that Bacillota and Pseudomonadota are common dominant phyla in dry-cured and fermented meat products and may participate in the transformation of substrates such as carbohydrates, amino acids, and lipids [29].

The bacterial communities in surface samples of Mianning ham were also mainly composed of Bacillota and Pseudomonadota. Among these phyla, Bacillota showed the highest relative abundance in surface samples of one-year-aged ham, accounting for 87.16%; its relative abundances in surface samples of two-, three-, and four-year-aged hams were 48.75%, 54.90%, and 75.40%, respectively. Unlike in internal samples, Bacillota maintained a relatively high abundance across all ripening years in surface samples and remained the dominant phylum. Pseudomonadota had the highest relative abundance in surface samples of two-year-aged ham, at 48.10%, and then decreased to 18.64% in four-year-aged ham. Bacteroidota and Actinomycetota were present at relatively low abundances in all surface samples.

At the genus level, the bacterial community composition in internal samples of Mianning ham was relatively complex and mainly included *Staphylococcus*, *Kushneria*, *Peptoniphilus*, and *Anaerococcus*. *Staphylococcus* showed relatively high abundance in internal samples across all ripening years, with relative abundances of 15.70%, 7.73%, 20.91%, and 14.77% in one-, two-, three-, and four-year-aged ham samples, respectively. It was one of the dominant genera in internal samples of one- and three-year-aged hams. *Kushneria* showed relative abundances of less than 3% in internal samples of one- to three-year-aged hams, whereas its abundance increased to 23.84% in internal samples of four-year-aged ham, making it one of the dominant genera in four-year-aged ham. Bacteria of the genus *Kushneria* generally exhibit strong salt tolerance and have been detected in various high-salt environments and salted foods [30]. In addition, studies have suggested that some halotolerant bacteria may inhibit the growth of undesirable microorganisms through competitive interactions and influence the formation of harmful metabolites such as amines [31]. The relative abundances of *Peptoniphilus* and *Anaerococcus* gradually decreased with increasing ripening time, from 14.10% and 8.78% in one-year-aged ham to 0.51% and 0.15% in four-year-aged ham, respectively. *Peptoniphilus* can transform proteins and carbohydrates into volatile fatty acids, carbon dioxide, hydrogen, and other substances and may also provide substrates for electroactive bacteria or other microbial groups [32]. *Anaerococcus* has been reported to utilize lactate as an electron donor during carbon-chain elongation and to contribute to the production of short-chain fatty acids, especially butyric and caproic acids [33]. These metabolic activities may indirectly influence the accumulation of flavor-related compounds in ham.

At the genus level, the bacterial communities in surface samples of Mianning ham were mainly composed of *Staphylococcus*, *Bacillus*, and *Erwiniaceae_unclassified*. *Staphylococcus* was one of the dominant genera on the ham surface, but its relative abundance generally decreased with increasing ripening time, accounting for 84.42%, 45.03%, 38.32%, and 29.50% in surface samples of one-, two-, three-, and four-year-aged hams, respectively. *Bacillus* increased markedly in surface samples of four-year-aged ham, rising from a relatively low level during the first three years to 41.57%, and became the dominant genus in four-year-aged ham surface samples. *Bacillus* has also been reported as one of the dominant genera in Xuanwei ham [34]. Notably, *Erwiniaceae_unclassified* accounted for only 4.63% in one-year-aged ham samples, increased to 44.56% in the surface samples of two-year-aged ham, and then decreased year by year in the three- and four-year-aged ham samples. The relative abundances of *Cobetia* in the surface samples of one-, two-, three-, and four-year-aged hams were 3.53%, 0.39%, 3.99%, and 5.87%, respectively. Previous studies have shown that *Cobetia* may be associated with the formation of some key flavor compounds in Mianning ham and is significantly positively correlated with volatile flavor compounds such as octane, 3-methylthiopropanal, and trans-2-nonenal [8].

### 3.5. Analysis of Fungal Community Structure

#### 3.5.1. Analysis of Fungal Diversity

As shown in Table 3, 575,741 raw sequences were obtained by sequencing the fungal ITS1 region from Mianning ham samples at different ripening years. After paired-end sequence assembly, low-quality sequence filtering, and chimera removal, 567,271 effective sequences were obtained. Overall, the fungal sequencing data for all samples were of good quality and met the requirements for subsequent analysis of fungal community structure.

As shown in Table S2, in the internal samples of Mianning ham, the ACE and Chao1 indices were highest in two-year-aged ham, indicating that these samples had the greatest internal fungal community richness. The Shannon index of three-year-aged ham was significantly higher than those of the samples from the other ripening years (*p* < 0.05), and the Simpson index was also highest in these samples, suggesting that the three-year-aged samples had the highest internal fungal community diversity. Among the surface samples, the highest ACE and Chao1 indices were observed in the four-year-aged samples, reaching 86.20 and 86.48, respectively, indicating that these samples had the greatest surface fungal community richness. Meanwhile, the Shannon and Simpson indices of the surface samples from four-year-aged ham were also the highest (*p* < 0.05), at 1.70 and 0.49, respectively, suggesting that these samples had the highest surface fungal community diversity. Overall, fungal community richness was higher on the surfaces of the one- and four-year-aged samples than in their corresponding internal samples, whereas fungal richness was higher inside the two- and three-year-aged samples than on their surfaces.

As shown in Figure S1, for the internal Mianning ham samples, PCoA1 and PCoA2, explained 96% and 2% of the variation in fungal community structure, respectively, accounting for 98% of the total variation. The PERMANOVA showed significant differences in fungal community structure among internal samples from different ripening years (R^2^ = 0.9729, *p* = 0.001), indicating that ripening time significantly affected the internal fungal community structure of Mianning ham.

For the surface samples, PCoA1 and PCoA2 explained 78% and 12% of the variation in fungal community structure, respectively, accounting for 90% of the total variation. PERMANOVA (R^2^ = 0.7208, *p* = 0.015) showed significant differences in fungal community structure among samples from different ripening years, indicating that ripening duration was a major factor shaping the surface fungal community structure of Mianning ham.

#### 3.5.2. Fungal Community Composition

**Figure 8:**
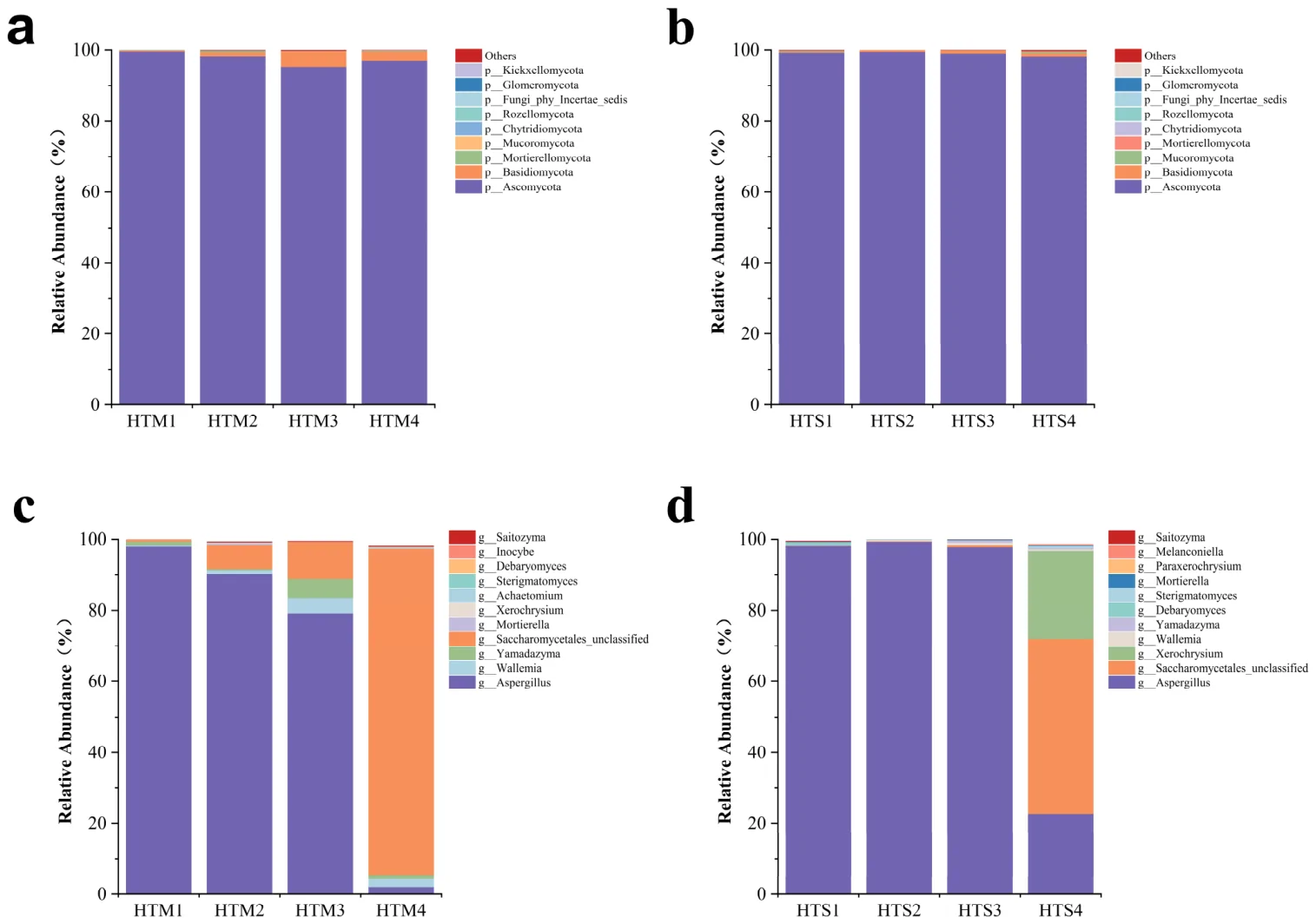
Stacked bar plots of the relative abundance of fungal communities at the phylum and genus levels. Notes: (**a**) internal fungal community at the phylum level; (**b**) surface fungal community at the phylum level; (**c**) internal fungal community at the genus level; (**d**) surface fungal community at the genus level.

Figure 8 shows that Ascomycota and Basidiomycota constituted the major fungal phyla in internal Mianning ham samples across the different ripening stages. Ascomycota accounted for 99.68%, 98.22%, 95.30%, and 97.02% of the fungal communities in the one-, two-, three-, and four-year-aged ham samples, respectively, and was dominant in all groups, indicating that it was the predominant fungal phylum in the internal fungal community of Mianning ham. This result is consistent with previous reports on the fungal community composition of traditional Chinese dry-cured hams, such as Xuanwei, Nuodeng, and Sanchuan hams, in which Ascomycota was also identified as the predominant fungal phylum [35]. The relative abundance of Basidiomycota in the internal samples was low, with the highest abundance observed in three-year-aged samples at 4.37%, and the lowest in one-year-aged ham samples, at 0.30%.

In the surface samples of Mianning ham, the fungal community was also dominated by Ascomycota, with relative abundances of 99.38%, 99.61%, 99.18%, and 98.16% in the one-, two-, three-, and four-year-aged ham samples, respectively. The relative abundance of Basidiomycota increased continuously with prolonged ripening, rising from 0.17% in the one-year-aged surface samples to 1.11% in the four-year-aged surface samples. These results indicate that Ascomycota was the dominant fungal phylum in both the internal and surface samples of Mianning ham, suggesting its stable dominance throughout ripening.

At the genus level, the internal fungal community of Mianning ham showed a clear succession pattern as ripening progressed. *Aspergillus* was the dominant genus in the internal samples from one-, two-, and three-year-aged hams, with relative abundances of 98.06%, 90.34%, and 79.32%, respectively. *Aspergillus* is commonly found in dry-cured and fermented foods. Some strains can produce proteases and lipases, which may participate in protein and lipid degradation and contribute to flavor formation [36]. With prolonged ripening, *Aspergillus* and *Saccharomycetales_unclassified* showed opposite abundance trends, with the former decreasing and the latter increasing gradually, reaching 91.78% in the internal samples from four-year-aged ham and becoming the dominant fungal group at this stage. This unclassified fungal taxon dominated the late ripening stage and may represent a characteristic fungal group in hams ripened for four years or longer; however, further investigation is needed. *Wallemia* and *Yamadazyma* showed initial increases followed by decreases in the internal samples, reaching their highest relative abundances in the three-year-aged samples, at 4.30% and 5.30%, respectively. These findings suggest that the internal fungal community of Mianning ham was dominated by *Aspergillus* during early ripening and gradually shifted toward yeast-related taxa during later ripening.

In the surface samples, *Aspergillus* was also the dominant genus in the one-, two-, and three-year-aged hams, with relative abundances of 98.23%, 99.45%, and 97.86%, respectively. However, in the surface samples from four-year-aged ham, the relative abundance of *Aspergillus* decreased to 22.69%. Meanwhile, the relative abundances of *Saccharomycetales_unclassified* and *Xerochrysium* increased markedly, reaching 49.33% and 24.76%, respectively, and these taxa became the major fungal groups on the surface of four-year-aged ham. *Wallemia* and *Yamadazyma* also showed initial increases followed by decreases in the surface samples, with their highest relative abundances observed in the three-year-aged samples, at 0.73% and 0.54%, respectively.

### 3.6. Correlation Analysis Between Microorganisms and Metabolites

To explore potential links between microbial succession and metabolite accumulation during Mianning ham ripening, 17 representative differential metabolites involved in significantly enriched KEGG pathways were selected for correlation analysis. Based on microbial relative abundance, the 20 most abundant genera were selected, and associations between microbial communities and metabolites were evaluated using Spearman correlation analysis.

**Figure 9:**
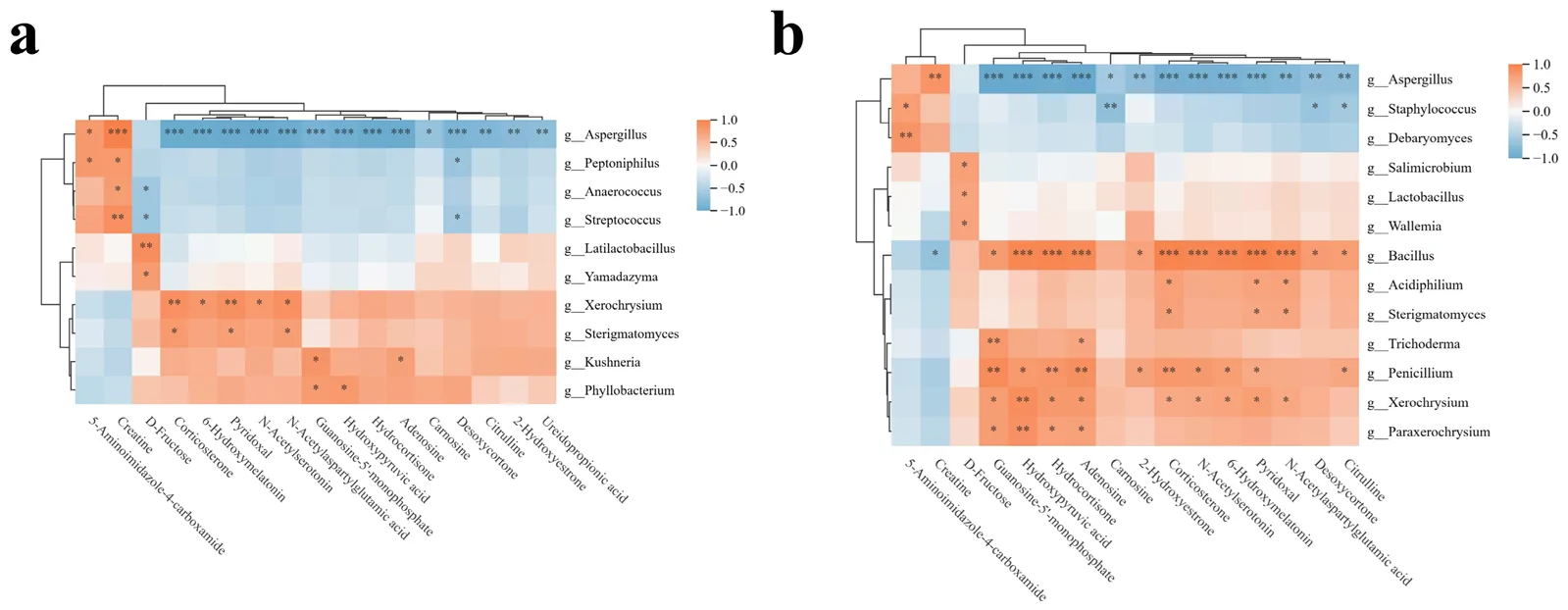
Correlation analysis between microorganisms and differential metabolites in Mianning ham. Notes: (**a**) correlations with internal microorganisms; (**b**) correlations with surface microorganisms. *, **, and *** indicate significant differences at *p* < 0.05, *p* ≤ 0.01, and *p* ≤ 0.001, respectively.

As shown in Figure 9a, internal microorganisms and differential metabolites displayed a clear clustered correlation pattern. Overall, the internal microbial taxa could be broadly divided into three groups. The first group included *Aspergillus*, *Staphylococcus*, and *Debaryomyces*. *Aspergillus* maintained a relatively high relative abundance during the ripening process of Mianning ham and was the dominant fungal genus in the microbial community. *Aspergillus* can produce various extracellular hydrolytic enzymes, including proteases and lipases, which participate in the degradation of protein and lipid components during meat ripening. In the present study, the differential metabolites significantly correlated with *Aspergillus* mainly included amino acid- and peptide-related metabolites, such as *N*-acetylaspartylglutamic acid and citrulline, suggesting that *Aspergillus* may be involved in protein degradation and the transformation of nitrogen-containing compounds during ripening.

The second group was represented by *Latilactobacillus* and *Yamadazyma*. Previous studies have shown that lactic acid bacteria can utilize carbohydrates and produce organic acids, thereby affecting the acidification environment during meat maturation [37]. D-fructose is a key metabolite involved in the fructose and mannose metabolism pathway. In the present study, its content reached a relatively high level in three-year-aged ham samples and showed a positive correlation with this microbial group. Among them, *Latilactobacillus* exhibited higher relative abundance in internal samples than in surface samples. These findings suggest that *Latilactobacillus* and *Yamadazyma* may participate in carbohydrate metabolism and transformation processes, thereby potentially affecting the internal maturation environment of Mianning ham.

The third group was represented by *Xerochrysium*, *Sterigmatomyces*, *Kushneria*, and *Phyllobacterium*. *Kushneria* is a halophilic bacterium that can adapt to high-salt and low-water-activity environments and utilize protein degradation products as nitrogen sources. During the ripening process of Mianning ham, *Kushneria* maintained a relatively high relative abundance in the fourth-year samples. Previous studies have shown that *Kushneria* increases rapidly during the late ripening stage of ham, which is consistent with the variation trend observed in the present study, suggesting that it may possess good adaptability to long-term ripening environments [30]. In the present study, *Kushneria* showed positive correlations with nucleoside- and nucleotide-related metabolites, including guanosine 5′-monophosphate (5′-GMP) and adenosine. These results suggest that *Kushneria* may participate in nucleotide-related metabolic processes. As an important umami nucleotide, 5′-GMP can enhance umami perception [38]. Therefore, *Kushneria* may influence the formation of umami-related compounds and flavor quality development in Mianning ham during the later stages of ripening by affecting purine metabolism-related processes.

As shown in Figure 9b, surface microorganisms and differential metabolites also exhibited a distinct clustered correlation pattern, although their association profile differed from that observed in internal samples. The first group was represented by *Aspergillus*, *Staphylococcus*, and *Debaryomyces*. *Staphylococcus* is a salt-tolerant bacterial genus frequently detected as a dominant microorganism in dry-cured ham, owing to its strong adaptability to high-salt environments during long-term ripening. Previous studies have shown that some *Staphylococcus* strains possess nitrate reductase, protease, and lipase activities, which contribute to color development, protein degradation, and lipid transformation during meat maturation [22,39]. Nitrate reductase activity promotes the formation of nitrite-related compounds, which are closely associated with the characteristic color development of cured meat products [40]. In the present study, *Staphylococcus* maintained a high abundance in surface samples, suggesting that it may play an important role in the formation of characteristic quality and flavor during the ripening of Mianning ham.

*Debaryomyces* can promote protein degradation and release free amino acids, such as valine, glutamic acid, arginine, and lysine. It may also enhance Strecker degradation, thereby generating aldehydes and other flavor-related compounds that improve the flavor of meat products [5]. These amino acids can serve as substrates for subsequent flavor compound formation, suggesting that *Debaryomyces* may participate in the initial transformation of protein-derived substrates during early ripening.

The second group, mainly including *Salimicrobium*, *Lactobacillus*, and *Wallemia*, showed a positive correlation with D-fructose. The third group was represented by *Bacillus*, *Penicillium*, *Xerochrysium*, *Paraxerochrysium*, and *Trichoderma*. *Bacillus* showed a significant increase in relative abundance in the fourth-year samples and became one of the dominant genera in the surface microbial community. The high-salt and low-water-activity conditions formed during long-term ripening may favor the colonization and persistence of *Bacillus* with strong environmental adaptability. Previous studies have shown that *Bacillus* exhibits strong protease activity, which can promote protein degradation and release small peptides and free amino acids, thereby contributing to the transformation of nitrogen-containing compounds during meat maturation [24]. *Bacillus* was positively correlated with several amino acid- and nitrogen-containing metabolites, including carnosine, citrulline, pyridoxal, and *N*-acetylaspartylglutamic acid, suggesting that it may participate in the further transformation of protein degradation products during late ripening. Previous studies have demonstrated that *Bacillus* can be used for the preparation of umami peptides from beef, indicating that this genus may promote the formation of flavor peptides and nitrogen-containing flavor precursors in ham, thereby contributing to the development of umami-related characteristics and flavor quality during late ripening [41].

In addition, *Xerochrysium* belongs to xerophilic fungal groups and exhibits strong adaptability to low-water-activity environments [42]. During the surface ripening process of Mianning ham, *Xerochrysium* showed a relatively high abundance and became a subdominant fungal genus in the fourth-year samples. Previous studies have shown that *Xerochrysium* is closely associated with multiple amino acid metabolic pathways [43]. *Xerochrysium* was positively correlated with several nitrogen-containing metabolites, suggesting that it may participate in the further transformation of protein degradation products and amino acid-related compounds during ripening, thereby influencing flavor quality development in Mianning ham during the later stages of maturation.

Overall, internal and surface samples exhibited distinct microorganism–metabolite association patterns during ripening. Changes in the internal microbial community were more closely associated with nucleotide-related metabolic alterations and the formation of umami-related characteristics. In contrast, surface microbial association patterns were more closely related to the transformation of protein-derived nitrogenous compounds and the accumulation of amino acid- and peptide-derived flavor precursors. These spatially differentiated association patterns suggest that internal microorganisms may be more involved in nucleotide-related metabolic processes, whereas surface microorganisms may be associated with the transformation of protein-derived flavor precursors. The coordinated changes between these microbial communities may contribute to the formation of the characteristic umami taste and mature flavor of Mianning ham. However, these results only reflect correlations between microorganisms and metabolites, and the specific underlying mechanisms require further validation.

## 4. Conclusions

The quality changes in Mianning ham during ripening were systematically characterized. As ripening progressed, moisture content and pH declined, whereas hardness, chewiness, and color changed significantly. Sensory flavor was markedly enhanced. Untargeted metabolomics indicated that ripening duration reshaped the small-molecule metabolic profile of Mianning ham primarily by promoting protein degradation, peptide release, and amino acid metabolism, driving a gradual transition from early substrate depletion to the enrichment of flavor precursors at later ripening stages. At the phylum level, Bacillota and Pseudomonadota dominated the bacterial communities in both surface and internal samples, whereas Ascomycota dominated the fungal communities. At the genus level, the internal samples were mainly characterized by *Staphylococcus* and *Kushneria*, whereas the surface samples were dominated by *Staphylococcus* and *Bacillus*. The fungal community gradually shifted from *Aspergillus* toward yeast-related taxa. Microbe–metabolite correlation analysis revealed distinct microorganism–metabolite association patterns between internal and surface samples of Mianning ham. Internal microbial communities were mainly associated with nucleotide metabolism- and umami-related characteristics, whereas surface microbial communities were more closely related to nitrogen-containing compound transformation and flavor precursor accumulation. These findings highlight the potential roles of microbial succession and metabolic remodeling in Mianning ham quality formation. Future studies combining functional validation of key microorganisms may further elucidate their mechanisms of interaction with flavor-related metabolites.

## Figures and Tables

**Table 1:** Color and texture properties of Mianning ham with different ripening periods.

Parameter	HT1	HT2	HT3	HT4
L*	29.67 ± 0.84 ^a^	21.91 ± 1.71 ^b^	17.33 ± 2.22 ^c^	13.98 ± 1.48 ^d^
a*	6.96 ± 1.35 ^b^	9.23 ± 1.43 ^b^	12.99 ± 1.72 ^a^	14.91 ± 1.07 ^a^
b*	8.75 ± 1.63 ^c^	11.35 ± 2.06 ^bc^	13.61 ± 1.01 a^b^	15.98 ± 0.78 ^a^
Hardness (gf)	1368.82 ± 73.52 ^d^	3101.65 ± 256.09 ^c^	8971.59 ± 75.26 ^b^	10,818.68 ± 827.57 ^a^
Springiness	0.40 ± 0.01 ^d^	0.50 ± 0.01 ^c^	0.67 ± 0.01 ^a^	0.54 ± 0.02 ^b^
Chewiness (gf)	269.14 ± 12.79 ^d^	869.80 ± 75.77 ^c^	3314.84 ± 245.82 ^a^	1914.49 ± 60.92 ^b^
Cohesiveness	0.48 ± 0.01 ^b^	0.57 ± 0.01 ^a^	0.38 ± 0.02 ^c^	0.56 ± 0.01 ^a^
Resilience	0.09 ± 0.00 ^c^	0.10 ± 0.00 ^b^	0.11 ± 0.00 ^a^	0.08 ± 0.00 ^d^

Notes: Different letter superscripts in the same row indicate statistically significant differences (*p* < 0.05).

**Table 2:** Statistics of bacterial 16S rRNA sequencing data.

Samples	Raw Reads	Valid Tags	Raw Bases (M)	Valid Bases (M)
HTM1	85,229	74,920	43	30
HTM2	84,205	67,485	42	27
HTM3	77,804	61,491	39	25
HTM4	77,976	61,798	39	25
HTS1	78,496	76,450	39	33
HTS2	73,449	71,963	37	31
HTS3	79,020	75,680	40	32
HTS4	77,104	74,887	39	32

Note: HTM1–HTM4 represent internal muscle samples from one- to four-year-aged hams, and HTS1–HTS4 represent surface samples from one- to four-year-aged hams.

**Table 3:** Statistics of fungal ITS sequencing data.

Samples	Raw Reads	Valid Tags	Raw Bases (M)	Valid Bases (M)
HTM1	71,488	70,328	36	18
HTM2	78,390	78,066	39	21
HTM3	70,357	69,655	35	19
HTM4	72,825	72,456	36	21
HTS1	74,029	73,232	37	19
HTS2	72,360	71,056	36	19
HTS3	65,407	64,549	33	17
HTS4	70,865	68,429	35	20

## Data Availability

The original contributions presented in this study are included in the article and supplementary material. Further inquiries can be directed to the corresponding authors.

## Supplementary Materials

The following supporting information can be downloaded at: https://www.mdpi.com/article/10.3390/foods15162831/s1. Figure S1. PCoA analysis of bacterial and fungal communities in ham samples. Note: (a) Internal bacterial community; (b) Surface bacterial community; (c) Internal fungal community; (d) Surface fungal community. Table S1. α diversity of the bacterial community in Mianning ham. Table S2. α diversity of the fungal community in Mianning ham.
