# Supplementary material for: Changes in Quality Characteristics, Microbial Communities, and Metabolomic Profiles of Mianning Ham During Different Aging Periods
Source: Foods. 2026 Aug 14;15(16):2831. doi: 10.3390/foods15162831 (PMC13512285; doi:10.3390/foods15162831)
Supplement: Supplementary file 1 [file foods-15-02831-s001.zip › foods-4472306-supplementary.pdf]

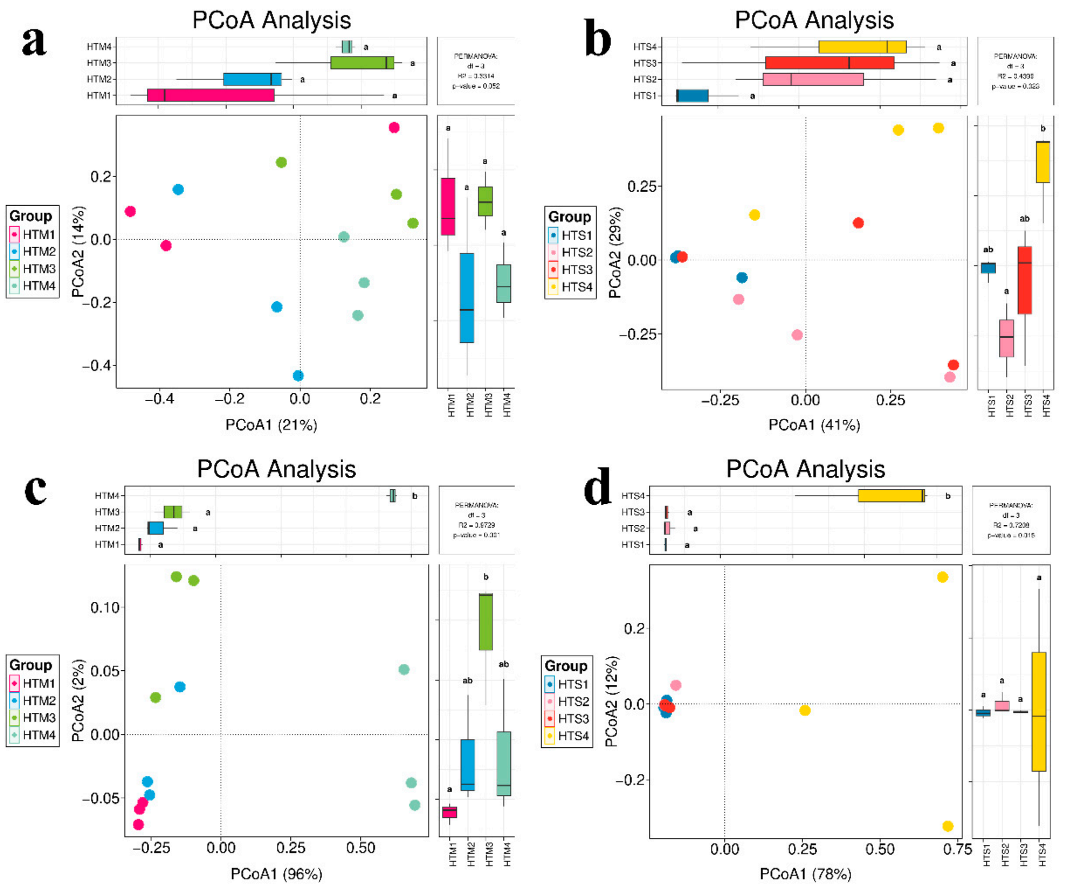

Figure S1. PCoA analysis of bacterial and fungal communities in ham samples. Note: (**a**) internal bacterial community; (**b**) surface bacterial community; (**c**) internal fungal community; (**d**) surface fungal community.

Table S1. *α* diversity of the bacterial community in Mianning ham.

**Samples**	**ace**	**Chao 1**	**Shannon**	**Simpson**	**goods_coverage**
HTM1	341.42±69.65^{ab}	328.68±66.49^{ab}	5.59±0.72^{a}	0.93±0.06^{a}	0.99
HTM2	157.17±161.12^{b}	152.80±154.01^{b}	5.06±0.77^{a}	0.95±0.02^{a}	0.99
HTM3	125.95±65.57^{b}	124.23±67.99^{b}	4.85±0.69^{a}	0.93±0.03^{a}	0.99
HTM4	104.54±44.27^{b}	104.17±47.40^{b}	4.38±1.86^{ab}	0.81±0.26^{ab}	0.99
HTS1	394.44±197.61^{ab}	394.74±206.96^{ab}	1.98±1.09^{c}	0.42±0.26^{c}	0.99
HTS2	266.13±57.85^{ab}	268.55±65.76^{ab}	2.18±0.42^{bc}	0.53±0.21^{bc}	0.99
HTS3	355.79±72.60^{ab}	357.39±63.80^{ab}	4.38±2.01^{ab}	0.75±0.25^{abc}	0.99
HTS4	499.71±322.25^{a}	488.56±321.25^{a}	3.65±1.08^{abc}	0.73±0.04^{abc}	0.99

Notes: Different letter superscripts in the same row indicate statistically significant differences ($p < 0.05$).

Table S2. α diversity of the fungal community in Mianning ham.

Samples	ace	Chao 1	Shannon	Simpson	goods_coverage
HTM1	22.06±10.61^{a}	20.94±10.01^{a}	0.34±0.07^{c}	0.07±0.02^{c}	0.99
HTM2	102.09±82.08^{a}	100.84±78.57^{a}	0.79±0.36^{bc}	0.20±0.12b^{c}	0.99
HTM3	68.61±36.84^{a}	71.39±40.25^{a}	1.39±0.30^{ab}	0.39±0.11^{ab}	0.99
HTM4	72.50±64.70^{a}	67.60±58.01^{a}	0.73±0.38^{bc}	0.16±0.09^{bc}	0.99
HTS1	56.67±37.26^{a}	58.96±34.45^{a}	0.34±0.13^{c}	0.07±0.03^{c}	0.99
HTS2	25.80±5.66^{a}	23.81±6.07^{a}	0.49±0.38^{c}	0.15±0.17^{bc}	0.99
HTS3	36.87±15.34^{a}	36.57±13.90^{a}	0.46±0.28^{c}	0.10±0.07^{c}	0.99
HTS4	86.20±31.17^{a}	86.48±28.91^{a}	1.70±0.85^{a}	0.49±0.29^{a}	0.99

Note: Different letter superscripts in the same row indicate statistically significant differences ($p < 0.05$).
